# Magnetically retainable microparticles for drug delivery to the joint: efficacy studies in an antigen-induced arthritis model in mice

**DOI:** 10.1186/ar2701

**Published:** 2009-05-19

**Authors:** Nicoleta Butoescu, Christian A Seemayer, Gaby Palmer, Pierre-André Guerne, Cem Gabay, Eric Doelker, Olivier Jordan

**Affiliations:** 1 School of Pharmaceutical Sciences, University of Geneva, University of Lausanne, Quai Ernest-Ansermet 30, 1211 Geneva, Switzerland; 2 Division of Pathology and Immunology, University Hospital of Geneva, Rue Michel-Servet 1, 1206 Geneva, Switzerland; 3 Division of Rheumatology, Department of Internal Medicine, University Hospital, Avenue Beau-Séjour 26, 1206 Geneva, Switzerland; 4 Department of Pathology and Immunology, University of Geneva School of Medicine, Rue Michel-Servet 1, 1206 Geneva, Switzerland

## Abstract

**Introduction:**

Conventional corticosteroid suspensions for the intra-articular treatment of arthritis suffer from limitations such as crystal formation or rapid clearance from the joint. The purpose of this study was to investigate an innovative alternative consisting of corticosteroid encapsulation into magnetically retainable microparticles.

**Methods:**

Microparticles (1 or 10 μm) containing both superparamagnetic iron oxide nanoparticles (SPIONs) and dexamethasone 21-acetate (DXM) were prepared. In a preliminary study, we compared the persistence of microparticles of both sizes in the joint. A second study evaluated the influence of a subcutaneously implanted magnet near the knee on the retention of magnetic microparticles in the joint by *in vivo *imaging. Finally, the efficacy of 10-μm microparticles was investigated using a model of antigen-induced arthritis (AIA) in mice. Phosphate-buffered saline, DXM suspension, SPION suspension, blank microparticles and microparticles containing only SPIONs were used as controls. Arthritis severity was assessed using ^99m^Tc accumulation and histological scoring.

**Results:**

Due to their capacity of encapsulating more corticosteroid and their increased joint retention, the 10-μm microparticles were more suitable vectors than the 1-μm microparticles for corticosteroid delivery to the joint. The presence of a magnet resulted in higher magnetic retention in the joint, as demonstrated by a higher fluorescence signal. The therapeutic efficacy in AIA of 10-μm microparticles containing DXM and SPIONs was similar to that of the DXM suspension, proving that the bioactive agent is released. Moreover, the anti-inflammatory effect of DXM-containing microparticles was more important than that of blank microparticles or microparticles containing only SPIONs. The presence of a magnet did not induce a greater inflammatory reaction.

**Conclusions:**

This study confirms the effectiveness of an innovative approach of using magnetically retainable microparticles as intra-articular drug delivery systems. A major advantage comes from a versatile polymer matrix, which allows the encapsulation of many classes of therapeutic agents (for example, p38 mitogen-activated protein kinase inhibitors), which may reduce systemic side effects.

## Introduction

The undeniable clinical efficacy of intra-articular (i-a.) corticosteroid injections is somehow restricted, on one hand, by the presence of crystals in the joint, possibly causing crystal-induced arthritis [1], and on the other hand, by the need for repeated injections, which can lead to joint instability [2] or infection [3]. Researchers thus have tried to encapsulate the corticosteroids into different drug delivery systems (that is, liposomes, nanoparticles and microparticles). Though more promising than steroid suspensions, these systems also faced a major drawback of short retention in the joint [4,5] due to the increased permeability of blood vessels in areas of inflammation [6].

To overcome these limitations, we investigated magnetically retainable drug delivery systems, an approach as yet clinically unexploited despite the intense need for the development of novel i-a. delivery modalities. Thus, our aim was to use biodegradable microparticles containing dexamethasone 21-acetate (DXM), from which the active substance could be slowly released during a well-defined period, avoiding the problem related to the appearance of crystals in the joint. The rapid clearance from the joint could possibly be overcome by co-encapsulating with DXM, superparamagnetic iron oxide nanoparticles (SPIONs). This would confer magnetic properties to the final microparticles, thus allowing their retention with an external magnetic field and possibly increasing their retention in the joint.

The first objective of this study was to choose the most suitable drug delivery system for the local treatment of joint inflammation. In this respect, we intra-articularly injected magnetic microparticles 1 or 10 μm in diameter and studied their retention at 3 months by histological analysis and *in vivo *imaging. The second objective was to determine the influence of a subcutaneously implanted magnet near the knee on the retention of microparticles in the joint. Finally, we studied the efficacy of microparticles containing DXM and SPIONs (referred to as complete microparticles) as an anti-inflammatory drug delivery system in an experimental model of antigen-induced arthritis (AIA) in mice.

## Materials and methods

### Microparticle preparation

**Figure 1:**
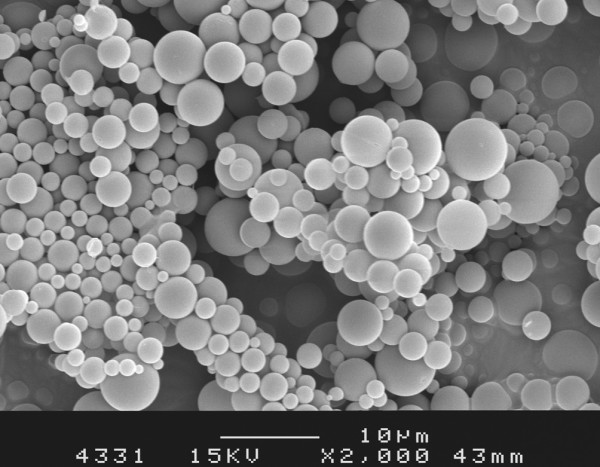
Scanning electron microscopy image of the microparticles.

**Figure 2:**
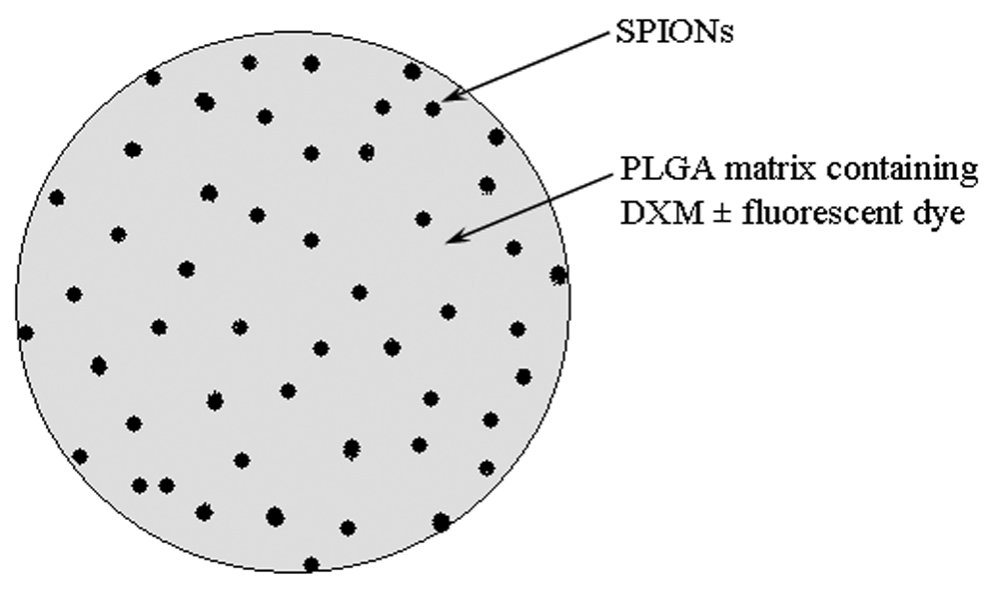
Schematic representation of a microparticle. DXM, dexamethasone 21-acetate; PLGA, poly(D, L-lactide-co-glycolide); SPION, superparamagnetic iron oxide nanoparticle.

The microparticles of a mean of 1 and 10 μm in diameter (Figure 1) were prepared using a double emulsion-solvent evaporation method in accordance with the protocol described by Butoescu and colleagues [7]; a schematic representation of a microparticle is presented in Figure 2. The polymer used as a matrix for the microparticles was poly(D, L-lactide-*co*-glycolide) (PLGA) with a molecular mass of 19 kDa (Resomer^® ^RG572S; Boehringer Ingelheim GmbH, Ingelheim, Germany). The diameter distribution of the 1-μm microparticle batch ranged from 0.4 to 1.4 μm and that of the 10-μm microparticle ranged from 4 to 14 μm. Blank microparticles were used as a control; the contents of DXM and SPIONs in the batches used as treatment were 2.5% and 1%, respectively. For the *in vivo *imaging experiment, microparticles were stained with fluorescent (near-infrared) NIR 780 phosphonate (λ_ex_/λ_em _= 640/825 nm) purchased from Fluka (Sigma-Aldrich, Buchs, Switzerland). The use of this dye allowed the detection of the microparticles at a wavelength in the NIR domain, where the autofluorescent background of fur and collagen is negligible.

### *In vivo *imaging

Sixteen healthy C57Bl/6 mice (Harlan, Horst, The Netherlands), 8 to 10 weeks old, were put under isofluorane anaesthesia and intra-articularly injected with 10 μL of a 3.6 mg (dry weight)/mL 10-μm microparticle suspension in sterile phosphate-buffered saline (PBS) while four mice were injected with PBS and used as controls. The microparticles were stained prior to injection with fluorescent NIR 780 phosphonate for imaging in the living animals. The left knee was intra-articularly injected with a microparticle suspension, whose quantity was chosen while keeping in mind that the DXM dose that needed to be delivered to the joint would be 1.2 mg/kg, according to El Hakim and colleagues [8]. Four days prior to the experiment, half of the mice were subcutaneously implanted with disc magnets on the external part of the left thigh, near the knee. The other half were used as magnet-free controls. The magnet implantation was verified by micro-computed tomography (CT) (Skyscan-1076; Gloor Instruments AG, Uster, Switzerland) on all animals. The acquired images were analysed with ImageJ software (National Institutes of Health, Bethesda, MD, USA) to determine the distance and angle between the magnet and the knee, thus permitting calculation of the magnetic flux density exerted on the injected microparticles for each mouse. The right knee was not injected. After injection, all animals were examined via *in vivo *fluorescence imaging (IVIS-200; Xenogen Corporation, Hopkinton, MA, USA) at days 1, 2, 3, 4, 7, 14 and 21. The image acquisition was done by using an indocyanine green filter, which allows the measurement of an excitation wavelength of 710 to 760 nm and an emission wavelength of 810 to 875 nm. The acquisition time was set at 3 seconds. The fluorescence intensity was expressed as the number of photons per second per square centimetre. At the end of the experiment, mice were sacrificed by CO_2 _inhalation and the knees were collected for histological analysis.

For the 3-month preliminary study on microparticle retention in the joint, four mice were used: two mice injected with 1-μm (mean diameter) microparticles and two with 10-μm (mean diameter) microparticles. Both knees were intra-articularly injected with a 3.6 mg/mL microparticle suspension. The left knee was implanted with a magnet and the right one was used as a magnet-free control. After 90 days, the mice were sacrificed by CO_2 _inhalation and the knees were collected for histological analysis.

### Antigen-induced arthritis

AIA was induced in male C57Bl/6 mice as previously described [9]. In brief, mice were immunised on day 0 via intradermal injection at the tail root with 100 μL of 2 mg/mL methylated bovine serum albumin (mBSA) (Fluka) emulsified 1:1 with Freund's complete adjuvant (Sigma-Aldrich), containing 1 mg/mL *Mycobacterium tuberculosis*. A second immunisation was performed on day 7 via intradermal injection of 100 μL of 2 mg/mL mBSA emulsified 1:1 with Freund's incomplete adjuvant (Sigma-Aldrich). On day 16 after the first immunisation, half of the mice were implanted on the external part of the left thigh, near the knee, with 1.2 T permanent disc magnets (4 mm in diameter and 2 mm in height; Maurer Magnetic AG, Grüningen, Switzerland), which produce a 0.14 T magnetic field at the articulation site. Arthritis was induced on day 21 by i-a. injection of 10 μL of 10 mg/mL mBSA in PBS in the right knee. This injection was done along the suprapatellar ligament directly into the joint cavity. Concomitantly with the arthritis induction, the different treatment regimens were started. The right knee was injected with PBS and served as a control. Other controls used in the experiment, in the presence or absence of a magnet, were blank microparticles, SPION-containing microparticles, DXM suspension and SPION suspension. The test drug delivery system consisted of 10-μm microparticles containing DXM and SPIONs ("complete microparticles"). Five animals were used for each group. Joint inflammation was quantified by measuring the accumulation of ^99m^Tc pertechnetate in the knee at days 1 and 4 after arthritis induction (MINI-assay type 6–20 H gamma counter; Uehlinger-Pfiffner AG, Schöftland, Switzerland). Thus, a dose of 10 μCi ^99m^Tc per mouse was subcutaneously injected in the posterior neck region. After 30 minutes, the accumulation of the isotope was measured by external gamma counting by positioning the mice on a custom-made lead platform in which a small opening allows specific counting of the knee region. The acquisition time was set at 10 seconds, and each knee was counted three times, with repositioning of the mouse in between the three measurements. The ratio of ^99m^Tc accumulation in the inflamed arthritic knee to ^99m^Tc uptake in the contralateral control knee was calculated. A ratio higher than 1.1 indicated joint inflammation. Mice were sacrificed 4 days after arthritis induction. Blood was withdrawn by cardiac puncture and was left to coagulate for at least 30 minutes prior to centrifugation at 4,000 revolutions per minute to collect the serum. The knees were dissected, fixed with 4% formaldehyde in PBS and used for histological analysis. All experimental procedures on animals reported in this paper were performed in compliance with Swiss federal law on the protection of animals and in accordance with a protocol approved by the animal ethical committee of the Geneva University School of Medicine and the canton of Geneva authority (Direction Générale de la Santé, authorisation number 1084/3326/2).

### Histology

After fixation in 4% formalin, all knee joints were cut in the sagittal direction. After decalcification and embedding in paraffin, 4-μm sections were cut and stained with haematoxylin and eosin, Elastica van Gieson, Masson tri-chrome, toluidine blue and Pearl's Prussian blue to detect the presence of iron using light microscopy. Histological sections were graded by a pathologist (CAS) in a blinded manner. Cartilage erosion and joint destruction as well the intensity of inflammation, including 'pannus' formation, were scored in accordance with the method of Camps and colleagues [10], using a score ranging from 0 to 4 (0 = normal, 1 = minimal, 2 = moderate, 3 = severe and 4 = very severe). In addition, the relative amount of polynuclear neutrophils as part of the inflammation or pannus formation was assessed with a score also ranging from 0 to 4 (0 = no neutrophils present and 4 = maximal neutrophilic infiltration).

### Anti-bovine serum albumin antibody measurement in the mouse serum

Ninety-six-well plates (Maxisorp™; Nunc A/S, a brand of Thermo Fischer Scientific, Roskilde, Denmark) were coated overnight at 4°C with 1% BSA in PBS. Serially diluted mouse serum in 1% gelatin in PBS was added to each well and incubated for 2 hours at room temperature. Wells were washed four times with PBS added with 0.05% Tween 20 (PBST). Next, 100 μL of goat anti-mouse IgG-horseradish peroxidase (Santa Cruz Biotechnology, Inc., Santa Cruz, CA, USA) diluted 1:2,000 in PBST was added, and the plate was incubated for 1 hour at room temperature. The wells were washed with PBST and the colour was developed with 100 μL of 1:1 mixture of stabilised hydrogen peroxide and stabilised tetramethylbenzidine (substrate reagent pack; R&D Systems, Abingdon, UK). The reaction was stopped by adding 50 μL/well of 2N H_2_SO_4_. Plate reading was performed at 470 nm (Bio-Rad 550 Microplate Reader; Bio-Rad Laboratories, Inc., Hercules, CA, USA), and the results were expressed as the percentage of absorbance units of control mice.

### Magnetic flux density calculation

The flux density present at different distances from the magnet was calculated by using the electromagnetic modelling software ViziMag (Webskel, Ayrshire, UK).

### Statistical analysis

The Mann-Whitney test (Wilcoxon rank sum test) for unpaired variables was used to compare differences between groups with a non-Gaussian distribution. The Student *t *test was used to compare groups with a Gaussian distribution. A *P *value of less than 0.05 was considered significant. The data were expressed as the mean ± standard deviation.

## Results

### Magnet implant visualisation by micro-computed tomography scan

**Figure 3:**
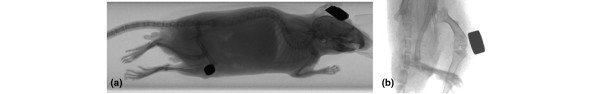
Micro-computed tomography images of magnet implantation. **(a) **Mouse scan. **(b) **Detail of the knee joint region that served for measuring the distance between the magnet and the knee.

All animals implanted with a magnet and used either for the *in vivo *imaging experiment or for the efficacy testing in the AIA model were imaged by micro-CT scan in order to assess the magnet location. A model of the acquired image is presented in Figure 3. These images allowed the calculations of the distance between the magnet and the knee and of the magnetic flux density exerted on the microparticles in the joint, using ViziMag software. Moreover, we determined that the magnetic flux density did not dramatically change with the angle to the magnetisation axis, remaining at around 0.1 T for angles between 0° and 90°. In contrast, the flux density rapidly changed with the distance between the magnet and the knee. The measured mean distance was 6.5 ± 1.0 mm, corresponding to a flux density of 136 ± 54 mT.

### Comparative persistence of 1- and 10-μm microparticles

**Figure 4:**
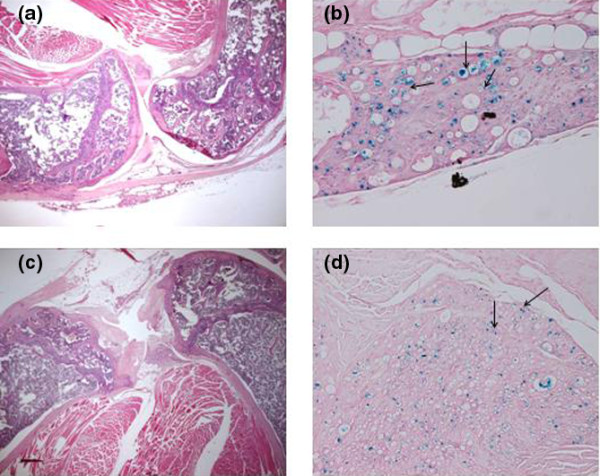
Histology of mouse knee joints 3 months after intra-articular injection of either 10-μm microparticles **(a, b)** or 1-μm microparticles **(c, d)**. Of note, even after 3 months, both types of microparticles are present in the tissue surrounding the joint cavity. Prussian blue (PB) staining provides evidence of iron within the microparticles; see arrows in (b, d). No major signs of inflammation are evident. Original magnifications: × 20 (a, c), × 400 (b) and × 100 (d). Stains: haematoxylin and eosin (a, c) and PB (b, d).

To identify the most suitable microparticle size to be used in the local treatment of arthritis, the articular retention of magnetic microparticles of a mean of 1 and 10 μm in diameter with and without a magnet was compared by means of *in vivo *imaging. For this long-term study, we used a dye covalently bound to the polymer chain PLGA-tetramethylrhodamine. For technical reasons, the magnet was maintained during only the first month. Although a visual difference in the presence and absence of a magnet can be noted in the acquired *in vivo *images, the fluorescence intensities were in the same order of magnitude (that is, the individual values obtained for each mouse at 75 days for 10-μm microparticles without a magnet were 2.33 × 10^5 ^and 2.69 × 10^5 ^and with a magnet were 3.10 × 10^5 ^and 3.37 × 10^5^). Nevertheless, a trend toward the improvement of microparticle retention in the presence of a magnet can be observed. The histological images (Figure 4) show that both 1- and 10-μm microparticles are still present in the joint 3 months after the injection and generated no inflammatory response or damage to the synovial lining.

### Influence of magnetic field on microparticle retention

**Figure 5:**
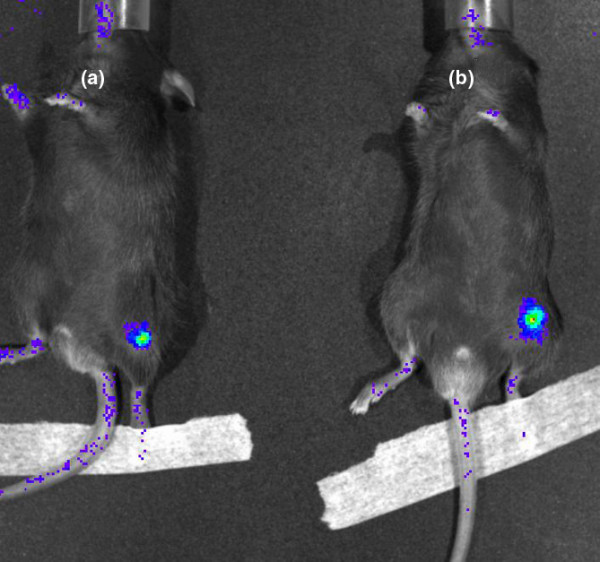
*In vivo *image obtained at 4 days after the intra-articular injection of fluorescent microparticles in the mouse knee joint without a magnet (mouse a) and with a magnet (mouse b).

**Figure 6:**
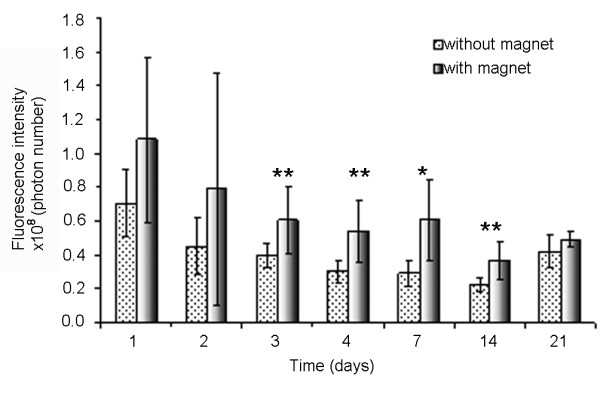
Fluorescence intensity in the presence or absence of a magnet. This graph shows a statistically significant difference by the presence of a magnet, which could have resulted from improved microparticle retention with magnet implantation (n = 8 mice per group). *P < 0.05; **P < 0.01.

Based on their good joint retention as demonstrated by the preliminary study and considering the fact that they can incorporate more DXM and SPIONs than the 1-μm microparticles, we chose the 10-μm microparticles for further therapeutic application. The next step was to determine the influence of an external magnet on the i-a. retention of this type of carrier by *in vivo *imaging. Figure 5 is an example of an image acquired with this technique. The fluorescent dye used to stain the microparticles has the advantage of absorption and emission wavelengths in the NIR domain, ensuring an optimal fluorescence/background signal ratio. Moreover, due to its small molar mass, it starts to slowly diffuse out of the microparticles after about 25 days (*in vitro *results not shown), which limited the duration of the study to 21 days. The plot of the fluorescence intensity versus time (Figure 6) demonstrates a signal decrease for groups with or without a magnet. Nevertheless, the signal reduction seemed to be less marked when a magnet was present. The differences between the two groups at days 3 to 14 are statistically significant, with *P *values ranging from 0.008 to 0.05, respectively (Mann-Whitney test). The increase in fluorescence intensity registered at day 21 could be due to a release of the encapsulated fluorescent marker, resulting from the degradation of the microparticle polymer matrix. The histological analysis of the knee joints confirmed the presence of microparticles positive for Prussian blue stain in the knees of the animals with or without a magnet, but no difference in the number of stained particles was observed visually.

### Efficacy of microparticles in antigen-induced arthritis in mice

To confirm that all mice were correctly immunised to mBSA, the levels of anti-mBSA antibodies were measured by enzyme-linked immunosorbent assay. All of the groups presented an adequate immune response of the levels of anti-mBSA antibody in the serum compared with an AIA-positive control mouse, with values generally superior to 80% of the positive control.

To determine the anti-inflammatory action of microparticles embedding DXM and SPIONs compared with controls, the accumulation of ^99m^Tc in the knee joints was measured at days 1 and 4 after i-a. injection. The values obtained at day 4 are expressed as the ratio of the gamma-counting values in the treated joint (left knee) and the untreated joint (right knee) (Tables 1 and 2). Data at day 1 were comparable with those obtained at day 4. In the animals treated with PBS, SPION suspension, blank microparticles and microparticles containing only SPIONs, the ^99m^Tc accumulation ratio had values of generally higher than 1.5, with a maximum of 2.2, reached for PBS-treated animals in the presence and absence of a magnet at days 1 and 4 after injection. In groups treated with DXM suspension and microparticles embedding DXM and SPIONs, a diminution of the inflammation was noted throughout the duration of the experiment. For example, at day 4 after injection, the values of the ^99m^Tc uptake ratio for animals treated with DXM suspension were 1.27 ± 0.17 in the group without a magnet and 1.21 ± 0.23 in the group with a magnet, but animals treated with the microparticles embedding DXM and SPIONs were 1.16 ± 0.1 without a magnet and 1.42 ± 0.19 with a magnet. No statistically significant differences were observed for the values in the presence or absence of a magnet for all groups, demonstrating that the presence of an implanted magnet did not induce a higher ^99m^Tc accumulation compared with the magnet-free animals. Importantly, the complete microparticles had an anti-inflammatory effect that was significantly higher compared with that of microparticles embedding only SPIONs, both in the presence and absence of a magnet. Surprisingly, a reduction in the inflammation was noted for the groups receiving polymer microparticles or SPION suspension, both with and without a magnet. No real reason was hypothesised, but the variability of the animal model response and the rather small number of animals per group could partially explain this trend. This observation questions the reliability of the conclusions drawn from the ^99m^Tc uptake measurements with respect to histological analysis.

**Table 1:** ^99m^Tc accumulation values obtained at day 4 when no magnet was implanted

	mBSA + PBS	DXM suspension	Polymer microparticles	SPION suspension	Microparticles + SPIONs	Complete microparticles
	(-)	(-)	(-)	(-)	(-)	(-)
	2.01	0.96	1.68	1.48	1.89	1.09
	2.32	1.33	1.76	1.54	1.23	1.31
	2.21	1.36	1.33	1.33	2.08	1.09
	2.31	1.38	1.55	1.65	2.09	1.08
	2.1	1.31	1.47	1.23	1.85	1.21
Mean	2.19	1.27	1.56	1.45	1.83	1.16
SD	0.13	0.17	0.17	0.17	0.35	0.10

The values are expressed as the ratio of the gamma-counting values in the treated joint (left knee) to those of the untreated joint (right knee). (-) indicates groups without a magnet. DXM, dexamethasone 21-acetate; mBSA, methyl bovine serum albumin; PBS, phosphate-buffered saline; SD, standard deviation; SPION, superparamagnetic iron oxide nanoparticle.

**Table 2:** ^99m^Tc accumulation values obtained at day 4 when a magnet was implanted near the left knee

	mBSA + PBS	DXM suspension	Polymer microparticles	SPION suspension	Microparticles + SPIONs	Complete microparticles
	(+)	(+)	(+)	(+)	(+)	(+)
	1.95	0.86	1.98	1.7	2.17	1.29
	1.7	1.33	1.35	1.75	2.08	1.65
	1.55	1.08	1.61	1.89	2.17	1.43
	1.83	1.38	1.64	1.54	2.15	1.18
	2.12	1.39	1.86	1.44	2.22	1.54
Mean	1.83	1.21	1.69	1.66	2.16	1.42
SD	0.22	0.23	0.24	0.18	0.05	0.19

The values are expressed as the ratio of the gamma-counting values in the treated joint (left knee) to those of the untreated joint (right knee). (+) indicates groups with a magnet. DXM, dexamethasone 21-acetate; mBSA, methyl bovine serum albumin; PBS, phosphate-buffered saline; SD, standard deviation; SPION, superparamagnetic iron oxide nanoparticle.

**Figure 7:**
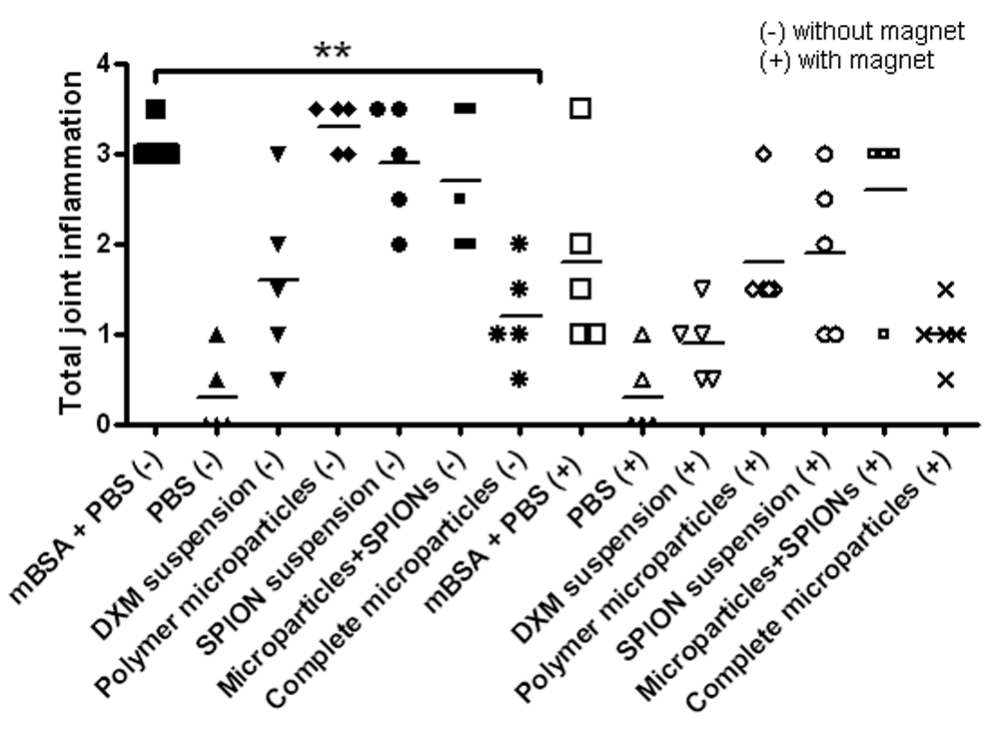
Histological grading of the knee sections for the total joint inflammation using a scale ranging from 0 to 4. (-) indicates groups without a magnet, and (+) indicates groups with a magnet. Results are expressed as individual values, and the horizontal line represents the mean (n = 5 mice per group). **P < 0.05 was considered significant. The histological analysis shows that the complete microparticles induced a significant inflammation reduction compared with the positive controls. The influence of the magnet on the inflammation score of the complete microparticle group is not significant. DXM, dexamethasone 21-acetate; mBSA, methyl bovine serum albumin; PBS, phosphate-buffered saline; SPION, superparamagnetic iron oxide nanoparticle.

The histological features of the knee joints of the test and control mice at day 4 after the i-a. injection confirmed that the presence of a magnet neither induces a higher inflammatory response nor leads to more marked cartilage erosion than in the magnet-free mice. Moreover, though not statistically significant, a trend toward the reduction of joint inflammation and cartilage damage in the presence of a magnet was noticed, especially for the groups treated with complete microparticles (Figure 7). This may be due to a high local microparticle concentration, leading to DXM release in the articular and periarticular zones and resulting in the diminution of inflammation. The use of five mice per group, a rather small number when considering the variability associated with the AIA experimental model, was compensated by the large number of screened conditions, thus providing new information on the effect of PLGA microparticles or SPION-containing microparticles on the synovial cavity. The total joint inflammation was significantly diminished in the group treated with complete microparticles in comparison with the PBS control (without a magnet), which (together with the absence of deleterious effects) proves the efficacy of our system. The histological scoring of the cartilage damage after the treatment with different products showed no or only slight erosion 4 days after arthritis induction (data not shown).

**Figure 8:**
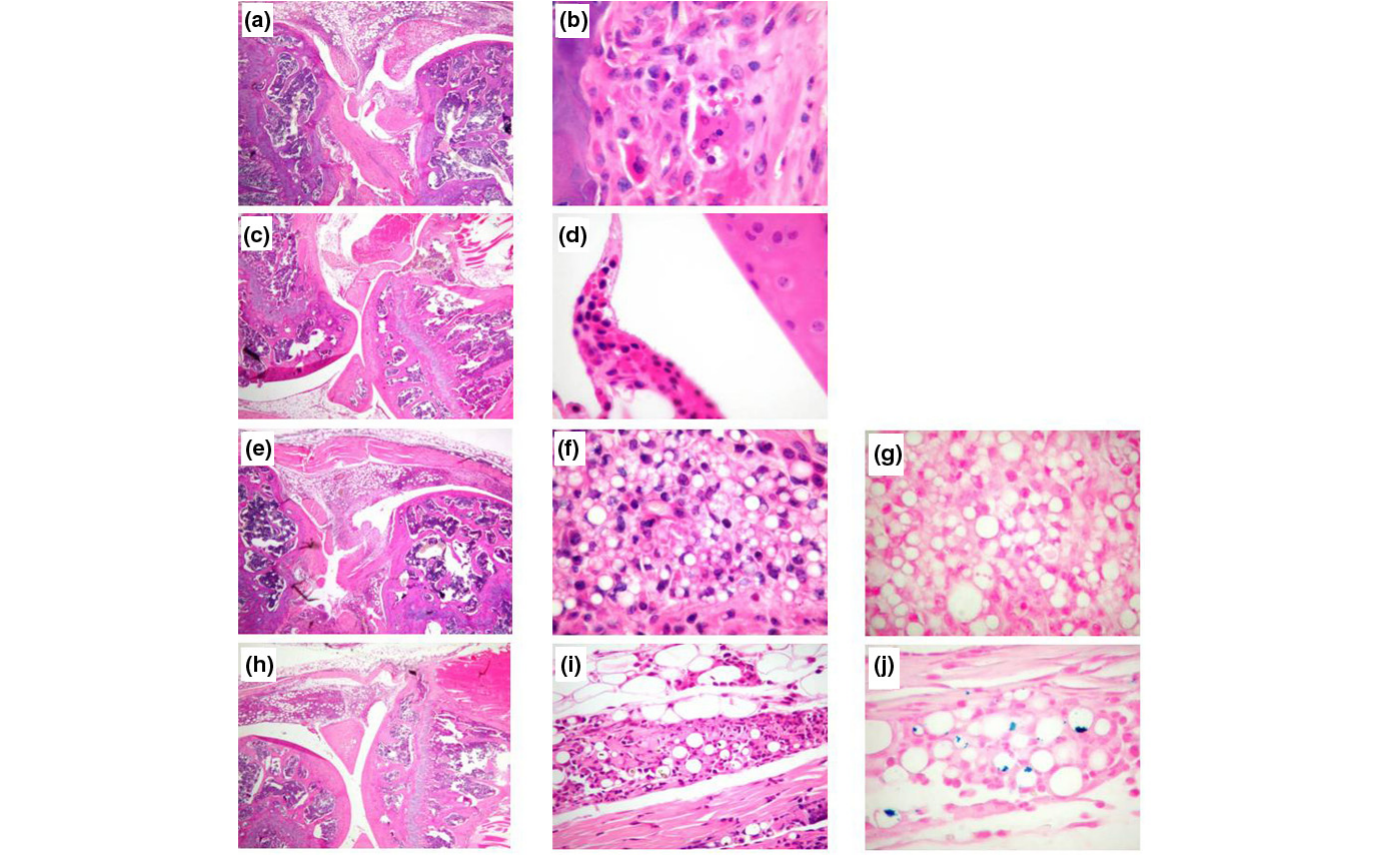
Histology of mouse knee joints 4 days after intra-articular injection. Staining is with haematoxylin and eosin unless specified otherwise. **(a, b) **Antigen-induced arthritis (AIA), positive control. (a) Intense inflammatory infiltrate in the synovial tissue and the joint cavity. (b) At a higher magnification, mononuclear inflammatory cells destroyed cartilage and modulated bone. **(c, d) **Negative control, phosphate-buffered saline. No inflammatory infiltrate is present either in the synovial tissue or the joint cavity. The cartilage surface is smooth. **(e-g) **AIA knees treated with microparticles without iron or dexamethasone 21-acetate (DXM). (e) Pronounced inflammatory infiltrate and cartilage destruction by a synovial 'pannus'. (f) Presence of numerous microparticles in synovial macrophages mixed with some polynuclear cells. (g) Prussian blue (PB) staining without evidence of iron. **(h-j) **AIA knees treated with microparticles containing iron and DXM. A reduction of inflammation in the synovial tissue is apparent when compared with (e-g). (h) No inflammation of the joint cavity or cartilage invasion or bone destruction is apparent. (i, j) Presence of microparticles in macrophages of the synovial tissues containing iron (j, PB). Original magnifications: × 20 (a, c, e, h) and × 400 (b, d, f, g, i, j).

Frames a and b of Figure 8 display histological images of a positive control knee, where signs of inflammation and synovial hyperplasia were present, in contrast to a negative control animal (Figure 8c, d), for which the knee joint showed no histological abnormality. The images corresponding to blank microparticle-injected joints (Figure 8e–g), similarly to the positive control mice, demonstrated focal accumulation of macrophages in the synovial space as well as in the periarticular zone. Moreover, the Prussian blue staining was negative, revealing the absence of SPIONs in the particles. The images of the mice knee joints treated with complete microparticles (Figure 8h–j) presented only minor signs of inflammation, thus demonstrating that the active substance was locally released and acted against the symptoms of arthritis. Microparticles were taken up mainly by the macrophages, which positively contributed (along with the magnet) to their retention in the joint. In addition, we performed an immunohistochemical reaction with macrophage-specific anti-MAC2 antibody and demonstrated that the cells containing the microparticles were macrophages (images not shown). Moreover, the Prussian blue staining was positive, indicating that the SPIONs were still embedded in the microparticles. Thus, this histological analysis, performed on the knees of all of the animals 4 days after the injection, validated the macroscopic observations as well as the results obtained for the uptake of ^99m^Tc.

## Discussion

To address the shortcomings related to the intra-articularly administered DXM suspension, we investigated the clinical potential of a novel system, namely magnetically retainable biodegradable microparticles gradually releasing DXM, for the local treatment of arthritis. The magnetic properties of this system come from the encapsulated SPIONs, which are nearly identical to the iron oxide used as a contrast agent in humans [11-13]. Using healthy mice, we addressed the possible SPION local toxicity in a previous study [14] and found that the i-a. injection did not lead to synovial inflammation. Moreover, we expected no systemic toxicity related to SPION presence in the joint due to the fact that the SPION quantity used in microparticles was 20- to 30-fold smaller than that used as contrast agent and that they were locally administered. Furthermore, the i-a. DXM dose used in the present study in mice was proportional to that currently used in humans. SPIONs and DXM were embedded in a biodegradable polymer matrix consisting of PLGA with a molecular weight of 19 kDa, resulting in an *in vivo *DXM sustained release throughout 6 days, as assessed by a dorsal air pouch model in mice [15].

To choose the most suitable microparticle size in terms of injectability, retainability in the joint in the absence or presence of a magnet and lack of proinflammatory activity, we performed a preliminary study on mice. This study revealed that both 1- and 10-μm microparticle suspension i-a. injections in healthy mice did not lead to any major inflammatory response. In addition, the presence of an external magnet seems to be favourable to the persistence of both particle sizes in the joint, thus supporting our initial hypothesis. Moreover, the histological observations showed that particles were still present in the synovial cavity at 3 months after the injection, confirming that this drug delivery system could be valuable for targeting other anti-inflammatory substances, such as tumour necrosis factor-alpha or p38 mitogen-activated protein kinase (MAPK) inhibitors [16-18]. Nevertheless, for technological reasons, such as the encapsulation of larger DXM and SPION quantities, we preferred the 10-μm microparticles for further experimentation and future clinical application. Their magnetic retention, investigated in an extended *in vivo *imaging animal study on 16 mice, demonstrated that a disc magnet placed near the knee statistically improved their persistence in the joint for between 3 and 14 days. For longer periods, the difference between the groups with a magnet and those without a magnet became statistically insignificant, possibly due to the fact that macrophage action of clearing the joint outweighed the magnet retention. An alternative explanation could be related to the physical properties of the particles. In fact, the fluorescent dye may have started to diffuse from the microparticles more rapidly than the observed *in vitro *release rates (results not shown) due to the acidic medium in the lysosomes and to the presence of enzymes, resulting in a diminution of the *in vivo *fluorescence intensity. The magnetic field strength (flux density) of around 140 mT used in the *in vivo *experiments is in accordance with those generally used in mice or humans [19]. The histological analysis of the knees following the *in vivo *imaging study did not show any histological abnormalities or signs of inflammation or synovial hyperplasia, which reveal a good compatibility between the microparticles and the synovial tissues.

To determine the potential of DXM-containing magnetically retainable microparticles in i-a. diseases, we tested their efficacy in an experimental model of AIA in comparison with a large number of controls: PBS, DXM suspension, SPION suspension, blank microparticles and microparticles containing only SPIONs. This extended number of conditions, which led us to the use of a rather limited number of animals per group, was necessary for at least two reasons. First, there was a need to perform these control tests for the correct evaluation of the action of the complete microparticles. Second, due to the limited number of reports on intra-articularly injected SPION suspension [20] and the lack of reports on PLGA microparticles and SPION-containing microparticles, we decided to investigate the behaviour of these systems in the synovial cavity. The experiment proved that the administration of the drug-containing magnetic microparticles or of the other products did not result in any deleterious effect on the joint. Additionally, the presence of an implanted magnet had no harmful consequences on the synovial cavity, as demonstrated by ^99m^Tc uptake or histological grading of the arthritis.

The arthritis induction by mBSA was performed at the same time as the injection of the control and treatment products. An important technical aspect is that immunisation against mBSA correctly operates even in the presence of different microparticle types, as demonstrated by the signs of arthritis detected in different animal groups. The AIA study in mice revealed that microparticles containing DXM and SPIONs presented an efficacy as good as DXM suspension, proving, on one hand, that the active substance is released from the microparticles and reaches the corticoid receptors and, on the other hand, the success of the injection method. Furthermore, the difference between the groups treated with PBS and those with drug-containing magnetic microparticles was statistically significant both in terms of ^99m^Tc accumulation and total joint inflammation by histological grading. In addition, a better anti-inflammatory action of the complete microparticles compared with the DXM suspension was observed in the case of histological grading of the inflammation or of the cartilage erosion, but no statistical difference could be calculated. Contrary to our expectations resulting from the *in vivo *imaging study, which demonstrated increased fluorescence intensity in the presence of a magnet at day 4, the efficacy of complete microparticles in AIA did not significantly improve in the presence of a magnetic field. Nevertheless, a trend toward the reduction of both joint inflammation and cartilage erosion was observable in all groups of animals implanted with a magnet, a fact that was also supported by the histological analysis of the knee joint. For future experimentation, we should consider using a larger number of animals per group in conjunction with a reduced number of groups and monitoring the concentration of the active substance inside the joint cavity. Moreover, experiments using an osteoarthritis model over extended time periods will be more appropriate to describe the benefit brought by SPION incorporation.

## Conclusions

Following i-a. administration of microparticles containing DXM and SPIONs in arthritic joints, a diminution in the synovial inflammation was observed 4 days after the injection. Furthermore, magnetic microparticles were still detectable in healthy joints up to 3 months after i-a. injection, proving that this versatile type of system could be promising in encapsulating other substances into the same microparticle type while the release rate could be tailored by changing the material of the microparticle matrix. In this respect, new formulation strategies could be found for very active compounds (for example, p38 MAPK or interleukin-1-beta inhibitors [pralnacasan]), which due to systemic toxicity could not be used otherwise. In a future project, it might be of interest to investigate the effect of magnetic microparticles in chronic inflammatory animal models, such as osteoarthritis, in which the 3-month persistence of microparticles in the joint could represent a real benefit. Another perspective opened by this research consists of chemically or physically modifying the microparticles to permit them to reach specific target sites in the inflamed joint.

## Abbreviations

AIA: antigen-induced arthritis; BSA: bovine serum albumin; CT: computed tomography; DXM: dexamethasone 21-acetate; i-a.: intra-articular; MAPK: mitogen-activated protein kinase; mBSA: methyl bovine serum albumin; NIR: near-infrared; PBS: phosphate-buffered saline; PBST: phosphate-buffered saline with 0.05% Tween 20; PLGA: poly(D, L-lactide-co-glycolide); SPION: superparamagnetic iron oxide nanoparticle.

## Competing interests

The authors declare that they have no competing interests.

## Authors' contributions

NB and CAS helped to perform the experiments, design the study and draft the manuscript. GP, P-AG, CG and ED helped to design the study, participated in the analysis and interpretation of data and helped to critically review the manuscript. OJ helped to perform the experiments and design the study, participated in the analysis and interpretation of data and helped to draft and critically review the manuscript. All authors read and approved the final version of the manuscript.
